# The Cardiopulmonary Effects of Ambient Air Pollution and Mechanistic Pathways: A Comparative Hierarchical Pathway Analysis

**DOI:** 10.1371/journal.pone.0114913

**Published:** 2014-12-12

**Authors:** Ananya Roy, Jicheng Gong, Duncan C. Thomas, Junfeng Zhang, Howard M. Kipen, David Q. Rich, Tong Zhu, Wei Huang, Min Hu, Guangfa Wang, Yuedan Wang, Ping Zhu, Shou-En Lu, Pamela Ohman-Strickland, Scott R. Diehl, Sandrah P. Eckel

**Affiliations:** 1 Department of Chronic Disease Epidemiology, Yale School of Public Health, New Haven, Connecticut, United States of America; 2 Duke University, Nicholas School of the Environment and Duke Global Health Institute, Durham, North Carolina, United States of America; 3 University of Southern California, Keck School of Medicine, Department of Preventive Medicine, Los Angeles, California, United States of America; 4 Environmental and Occupational Health Sciences Institute, Rutgers Robert Wood Johnson Medical School, Rutgers University, Piscataway, New Jersey, United States of America; 5 University of Rochester, School of Medicine and Dentistry, Department of Public Health Sciences. Rochester, New York, United States of America; 6 Peking University, State Key Laboratory of Environmental Simulation and Pollution Control, College of Environmental Sciences and Engineering, and the Center for Environment and Health, Beijing, China; 7 Peking University, School of Public Health, Department of Occupational and Environmental Health and Institute of Environmental Medicine, Beijing, China; 8 Peking University First Hospital, Department of Pulmonary Medicine, Beijing, China; 9 Peking University Health Sciences Center, Department of Immunology, Beijing, China; 10 Peking University First Hospital, Department of Hematology, Beijing, China; 11 Rutgers School of Public Health, Department of Biostatistics, Piscataway, New Jersey, United States of America; 12 Rutgers School of Dentistry, Center for Pharmacogenomics and Complex Disease, Newark, New Jersey, United States of America; Nanjing University, China

## Abstract

Previous studies have investigated the associations between exposure to ambient air pollution and biomarkers of physiological pathways, yet little has been done on the comparison across biomarkers of different pathways to establish the temporal pattern of biological response. In the current study, we aim to compare the relative temporal patterns in responses of candidate pathways to different pollutants. Four biomarkers of pulmonary inflammation and oxidative stress, five biomarkers of systemic inflammation and oxidative stress, ten parameters of autonomic function, and three biomarkers of hemostasis were repeatedly measured in 125 young adults, along with daily concentrations of ambient CO, PM_2.5_, NO_2_, SO_2_, EC, OC, and sulfate, before, during, and after the Beijing Olympics. We used a two-stage modeling approach, including Stage I models to estimate the association between each biomarker and pollutant over each of 7 lags, and Stage II mixed-effect models to describe temporal patterns in the associations when grouping the biomarkers into the four physiological pathways. Our results show that candidate pathway groupings of biomarkers explained a significant amount of variation in the associations for each pollutant, and the temporal patterns of the biomarker-pollutant-lag associations varied across candidate pathways (p<0.0001) and were not linear (from lag 0 to lag 3: p = 0.0629, from lag 3 to lag 6: p = 0.0005). These findings suggest that, among this healthy young adult population, the pulmonary inflammation and oxidative stress pathway is the first to respond to ambient air pollution exposure (within 24 hours) and the hemostasis pathway responds gradually over a 2–3 day period. The initial pulmonary response may contribute to the more gradual systemic changes that likely ultimately involve the cardiovascular system.

## Introduction

Over the last decade there have been substantial inroads into understanding mechanisms involved in the cardiovascular effects of air pollution exposure [1], [2]. Epidemiological and animal studies indicate that exposure to air pollutants are linked to biomarkers of endothelial dysfunction, increased blood pressure, prothrombotic and coagulation changes, systemic inflammation and oxidative stress, autonomic imbalance, and arrhythmias [1], [3]. However, the mechanisms by which air pollutants exert their adverse effects on the cardiovascular system remain unclear. Currently, prominent hypotheses are that inhaled air pollutants can (1) initiate inflammatory response in the alveoli, which in turn trigger systemic inflammatory cascades resulting in cardiovascular effects; (2) be detected by afferent receptors within the respiratory tract which disrupt the balance of the autonomic system resulting in alterations in vascular tone and heart rate; (3) cross the blood-alveolar barrier and enter the systemic circulation where they directly affect the vasculature and alter hemostasis [1], [4].

The majority of existing studies investigated only a small number of biomarkers of specific physiological pathways. However, individual biomarkers in a specific pathway are under constant feedback regulation from bioactive molecules of other pathways; thus examining biomarkers in a specific physiological pathway might provide an incomplete snapshot of the underlying biology. In addition, studies of individual biomarkers are susceptible to overgeneralization to the whole pathway and publication bias, which makes it harder to determine the comparative effects of air pollutants on the different pathways involved in cardiovascular pathology. Consequently, the relative importance of the different pathways involved in the effects of air pollution on cardiovascular disease remains unclear.

In the present study, we utilize data on air pollution exposure and an extensive set of biomarkers collected in a panel study of healthy young adults followed through the 2008 Beijing Olympics, during which air pollution levels were drastically reduced [5], to quantify temporal patterns in the associations between pollutants and biomarkers of four candidate physiological pathways. Based on our previous findings from analyzing individual biomarkers, we hypothesized that biomarkers in the same candidate pathway would have similar temporal pattern in their responses to pollutant exposure [5], [6].

## Methods

### Study design and study population

A series of air pollution control measures were implemented from July 20 to September 17, 2008, encompassing the Olympic Games (August 8–24) through the end of the Paralympic Games (September 6–17). These control measures created the opportunity for a study design with ‘high-low-high’ pollution levels. Our study included three periods: (1) the pre-Olympic period (June 2–July 20) when light air pollution control measures were implemented, (2) the during-Olympic period (July 21–September 20) when industrial and commercial combustion facility operation and vehicle use were strictly controlled, and (3) the post-Olympic period (September 21–October 30) when the pollution control measures were relaxed [6], [7]. This panel study of air pollution and biomarkers of cardio-respiratory pathology was performed on the campus of Peking University First Hospital, Beijing (Latitude: 39.9272, Longitude: 116.3722).

We enrolled 125 young adult never-smokers who were free of cardio-respiratory, liver, kidney, neurologic, and other chronic diseases. Most study participants were medical residents working at the hospital and all participants lived within 9 km of the hospital. Participants were invited for clinical visits (between 8AM to 10AM) twice in each of the pre-, during-, and post-Olympic periods, in which the two visits were designed to be two weeks apart and at the same day of week. Participants were required to fast overnight before the clinical visits, refrained from taking any medications, working nightshifts or travelling, and were free of symptoms of respiratory infection or allergies within seven days prior to each clinical visit. The study population and data collection methods have been described in detail in previous publications [5]–[8].

This study was approved by the University of Medicine and Dentistry of New Jersey institutional review board and the joint Ethics Committee of the Peking University Health Sciences Center and the Peking University First Hospital. All participants provided written informed consent before participating in the study.

### Air pollution measurement

Air pollutants were monitored throughout all the three Olympic period (June 2-October 30, 2008). During these periods, we measured ambient concentrations of sulfur dioxide (SO_2_), nitrogen dioxide (NO_2_), ozone (O_3_), carbon monoxide (CO), fine particulate matter (PM_2.5_), and its constituents, elemental carbon (EC), organic carbon (OC), and sulfate (SO_4_^2−^); temperature and relative humidity (RH) were also recorded. Measurements were conducted on the roof of a seven-story building (∼20 meters above the ground) in the center of the hospital campus. We calculated average pollutant concentrations over seven-day periods before the time point where biological samples were collected according to the number of hours away from the sample collection (0–23 hours  =  lag 0, etc.). We excluded O_3_ from these analyses due to the strong negative correlation with other pollutants noted in our prior publications [5], [6], [8]. Additional description is provided in Supporting Information (S1 Appendix).

### Biomarker Measurements

We grouped the assessed biomarkers into 4 *a priori* candidate physiological pathways, including pulmonary inflammation and oxidative stress, autonomic function, hemostasis, and systemic inflammation and oxidative stress, based on biological activity and previous literature [5], [7]. We grouped inflammatory and oxidative stress biomarkers together because oxidative stress is often induced by and elicits inflammatory processes [1].

*Pulmonary inflammation and oxidative stress* were assessed using fractional exhaled nitric oxide (FeNO) and exhaled breath condensate (EBC) biomarkers, including pH value, nitrite, and malondialdehyde (MDA).

*Autonomic function* was assessed by systolic blood pressure (SBP), diastolic blood pressure (DBP), heart rate and heart rate variability (HRV), including standard deviation of normal R-R intervals (SDNN), root mean square of successive differences between adjacent normal cycles (rMSSD), low frequency (LF) power, high-frequency (HF) power, very low frequency (VLF) power, ratio of LF to HF, and total power.

*Hemostasis* markers included soluble P-selectin (sCD62P), CD40 Ligand (sCD40L), and von Willebrand Factor (VWF).

*Systemic inflammation and oxidative stress* markers included fibrinogen, red blood cells (RBC), white blood cells (WBC), and C-reactive protein (CRP) in plasma, as well as MDA and 8-Hydroxy-2′-deoxyguanosine (8-OHdG) in urine. CRP was excluded for these analyses due to a large number of non-detects (∼53%). Urinary concentrations of 8-OHdG and MDA were normalized by creatinine concentrations.

Additional description is provided in Supporting Information (S2 Appendix).

### Statistical analysis

**Figure 1:**
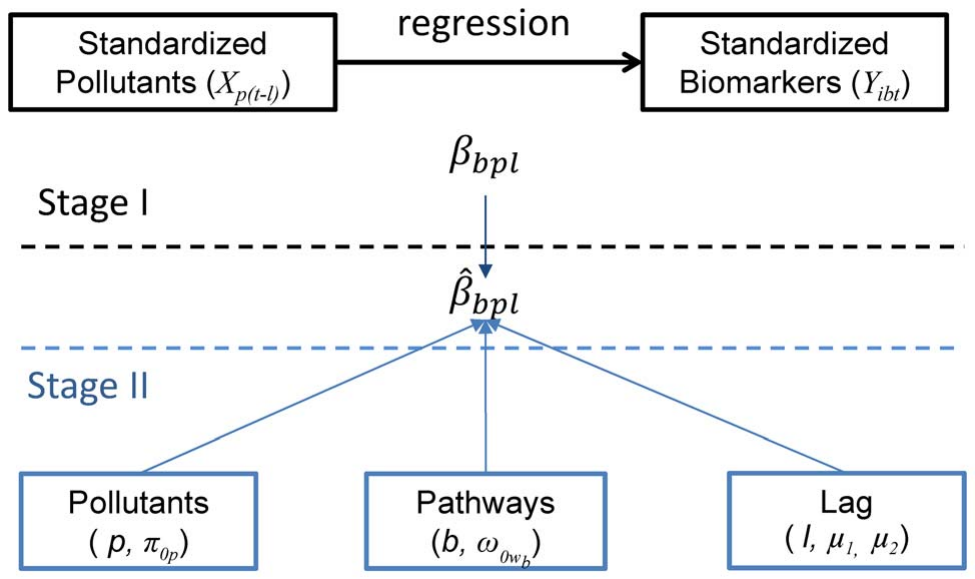
Conceptual framework of the hierarchical modeling approach.

Exploratory univariate and bivariate analyses were conducted to identify outliers and potential confounders of the relationships between biomarkers and pollutants. Values of EBC pH were multiplied by -1 so that higher levels would be considered a worse health condition for all biomarkers. Each biomarker and air pollutant level was internally standardized by [(*x_i_*-mean*_x_*)/SD_x_], where *X_i_* is each individual observation of a biomarker or pollutant, mean*_x_* and SD_x_ are grand mean and grand standard deviation of this biomarker or pollutant. We then developed and applied a two-stage statistical analysis (Fig. 1).

In Stage I, we used the mixed-effect models (Eq. 1) to estimate the association coefficients (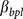) of a specific biomarker (*b*) with a specific pollutant (*p*) at a specific lag day (*l*). 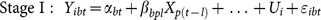
(Eq. 1)where 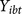 denotes the standardized value of biomarker *b* for participant *i* at visit *t*, 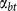 is the grand mean of biomarker *b* at visit *t*, 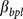 denotes the association coefficient of biomarker *b* with pollutant *p* at lag *l* at visit *t*, 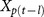 is the standardized concentration of pollutant *p* at lag *l* of visit *t*, and 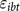 denotes the random error of the standardized concentration of biomarker *b* for participant *i* at visit *t*.

In these models, we adjusted for the following potential confounders (represented by ‘…’ in Eq. 1): sex, indicators of day of week, and smooth functions of temperature and relative humidity and included participant-level random intercepts (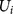) to account for repeated measurements on participants. Stage I model selection has been explained in detail previously [5], [6], [8]. Since the biomarkers and lagged pollutants were standardized, the Stage I, 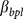 have similar interpretations, which facilitates comparison in Stage II. For any biomarker-pollutant-lag combination, 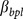 represents the difference in biomarker *b* associated with one standard deviation (SD) increase in pollutant *p* at lag *l*.

Stage II models were developed to explain variation in the temporally resolved biomarker-specific effects of each pollutant. Our statistical approach is an extension of repeated measures ANOVA. Specifically, Stage II consisted of a single linear mixed-effects model for 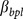 estimates (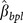) with inverse variance weighting to account for the wide range of standard errors (0.013 to 0.093) of 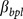: 
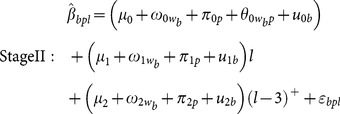
(Eq. 2)


In Eq. 2, differences in mean 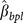 at lag 0 across pollutants are quantified by 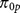 and differences in mean 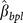 at lag 0 across pathways are quantified by 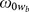 where 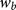 denotes the pathway to which biomarker *b* is assigned. For identifiability, for a reference pollutant (here PM_2.5_, denoted pollutant *p* = 1) and a reference pathway (here, systemic inflammation/oxidative stress, denoted pathway *w* = 1), 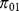 and 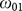 are both set to zero so that *µ_0_* quantifies mean 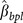 at lag 0 in the reference pathway and reference pollutant. Additional differences in mean 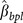 at lag 0 due to interactions between pathways and pollutants are quantified by *θ_0wp_*, with similar identifiability constraints for the reference pathway and pollutant. Biomarker-level random effects are represented by 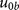, 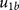, and 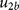. We specified an unstructured covariance matrix for the random effects and an autoregressive covariance matrix (AR-1) for the residuals as a function of lag to account for possible autocorrelation of 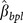 from the same biomarker-pollutant combination, across different lags.

**Figure 2:**
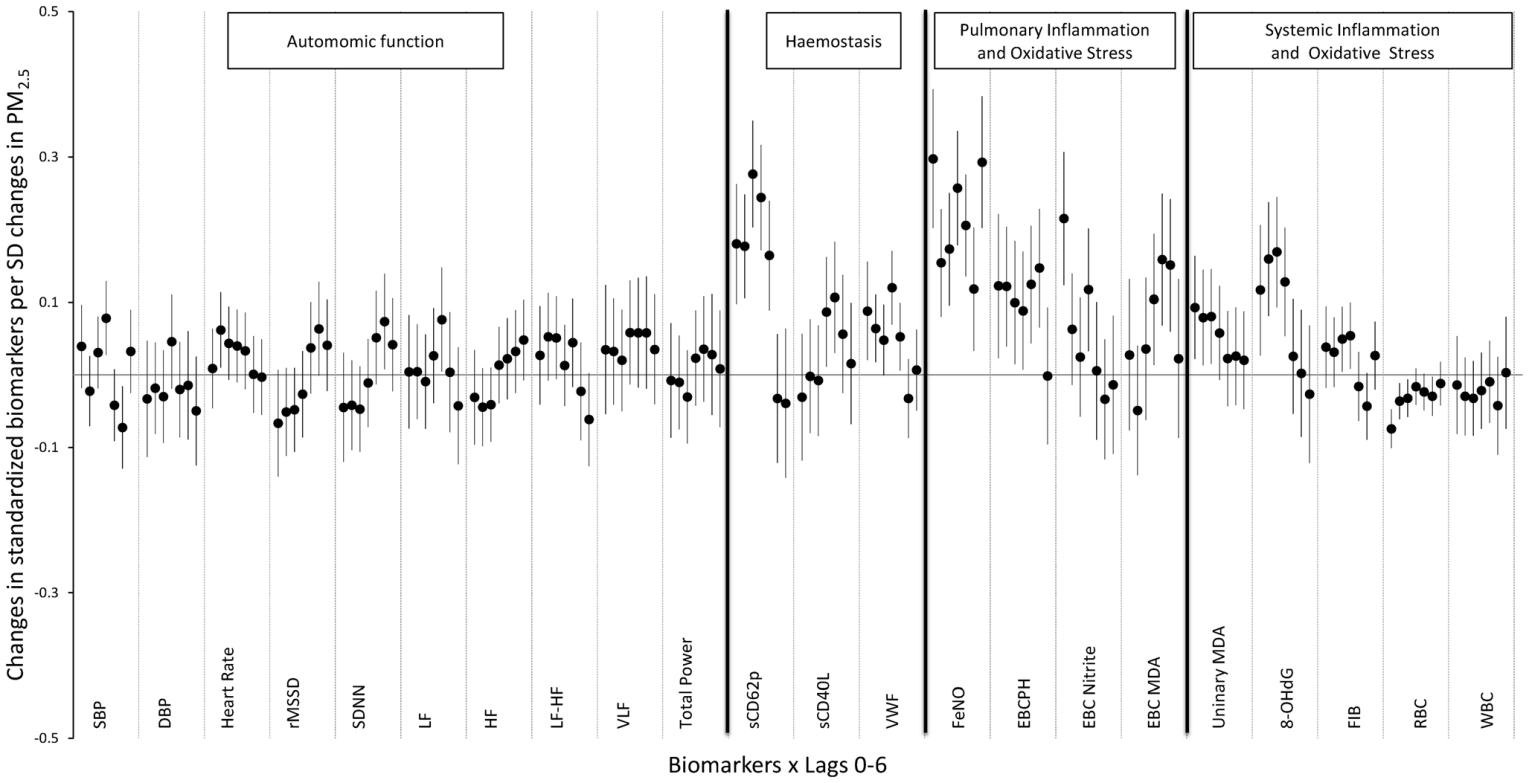
Associations (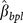) between standardized 24 hour average ambient PM_2.5_ concentrations and standardized biomarkers in each pathway, from Stage I models. Error bars represent 95% confidence intervals. Effect sizes are scaled to a 1 standard deviation change in PM_2.5_ (51.9 µg/m3).

Rather than assume a linear effect of lag on 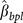, we used a piecewise linear spline with a change point (knot) in the middle of the 7 day period, at lag 3. This was a natural, not data-driven choice for the change point, and this approach offered a simple and reasonable representation of the patterns of association observed in Fig. 2. The spline is represented using two sets of terms, where the variable 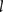 takes values 0, 1, 2, 3, 4, 5, 6 and 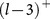 takes values 0, 0, 0, 0, 1, 2, 3. This relatively simple structure allowed us to investigate general patterns in the associations of biomarkers with each pollutant over 7 days, borrowing strength across biomarkers in the same pathway. For example, for ‘average’ biomarkers (where 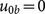, 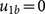, and 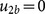) in the reference pathway (systemic inflammation/oxidative stress), the mean 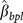 at lag 0 for the reference pollutant (PM_2.5_) is 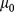, the daily rate of change in mean 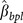 from lag 0 to lag 3 is 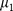 and the daily rate of change in mean 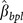 from lag 3 to lag 6 is 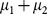 so that 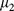 quantifies the difference in slopes between lags 0–3 and lags 3–6. As in a sensitivity analysis, we compared the AIC of our final model to that of otherwise identical models that used change points of 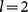 and 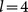
(1 and 5 were considered too close to the endpoints to be meaningful) and found that 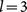
minimized AIC for the final model.

Overall, the grouping of biomarkers into physiological pathways allowed us to quantify and evaluate: (a) differences in associations at lag 0 across pathways (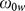) and across pollutants (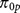), (b) whether pathway-level associations at lag 0 varied by pollutant (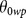), and (c) pollutant-specific, pathway-level temporal patterns of association (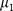
and 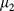, for the reference pathway and pollutant). For model selection, we evaluated evidence for a more complex model versus a more parsimonious model using likelihood ratio tests. We obtained predictions of 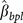 from the Stage II model using empirical Bayes predictions of the biomarker-level random effects.

Stage I statistical analyses were performed using the R Programming Language (Version 2.12.2; R Development Core Team) and Stage II analyses were performed using SAS (Version 9.3).

## Results

### Participant characteristics

The age of the 125 study participants were between 19 and 33 years old (mean: 24±2 years) with 63 male and 62 female subjects, as described in detail in previous publications [5], [6]. Among the enrolled participants, 119 (95.2%) finished all 6 visits and the other 6 (4.8%) completed 5 visits.

### Ambient air pollution levels

Air pollution concentrations in the three Olympic periods were reported previously [5], [7], and we included the period-specific mean and standard deviation of the seven pollutant concentrations in Supporting Information (S1 Table). Pollution levels declined substantially (13% to 60%) from the pre- to during-Olympic periods and increased substantially (21% to 197%) from the during- to post-Olympic periods, except for SO_4_^2−^ (declined 47%). At lag 0, the mean±SD for the 24 hour averaged pollutants were: 85.2±51.9 µg/m^3^ for PM_2.5_, 21.8±17.0 µg/m^3^ for SO_4_^2−^, 2.3±1.3 µg/m^3^ for EC, 10.2±6.6 µg/m^3^ for OC, 6.07±4.01 ppb for SO_2_, 0.91±0.5 ppm for CO, and 26.95±15.26 ppb for NO_2_.

### Stage I results

Fig. 2 displays 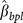 for PM_2.5_ (the estimated change in standardized biomarkers associated with a 1-SD increase in PM_2.5_ at each lag day, adjusting for sex, day of week, temperature, and RH. All biomarkers in the hemostasis and pulmonary pathways were positively associated with PM_2.5_ for at least one lag. The hemostasis biomarker associations generally increased and then decreased in magnitude over the 7 lag days. The pulmonary biomarkers had strong associations from lag 0. There appeared to be little association of biomarkers in the other pathways with PM_2.5_. The 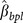 for all pollutants are summarized in Supporting Information (S2 Table).

### Selecting a parsimonious Stage II model

**Figure 3:**
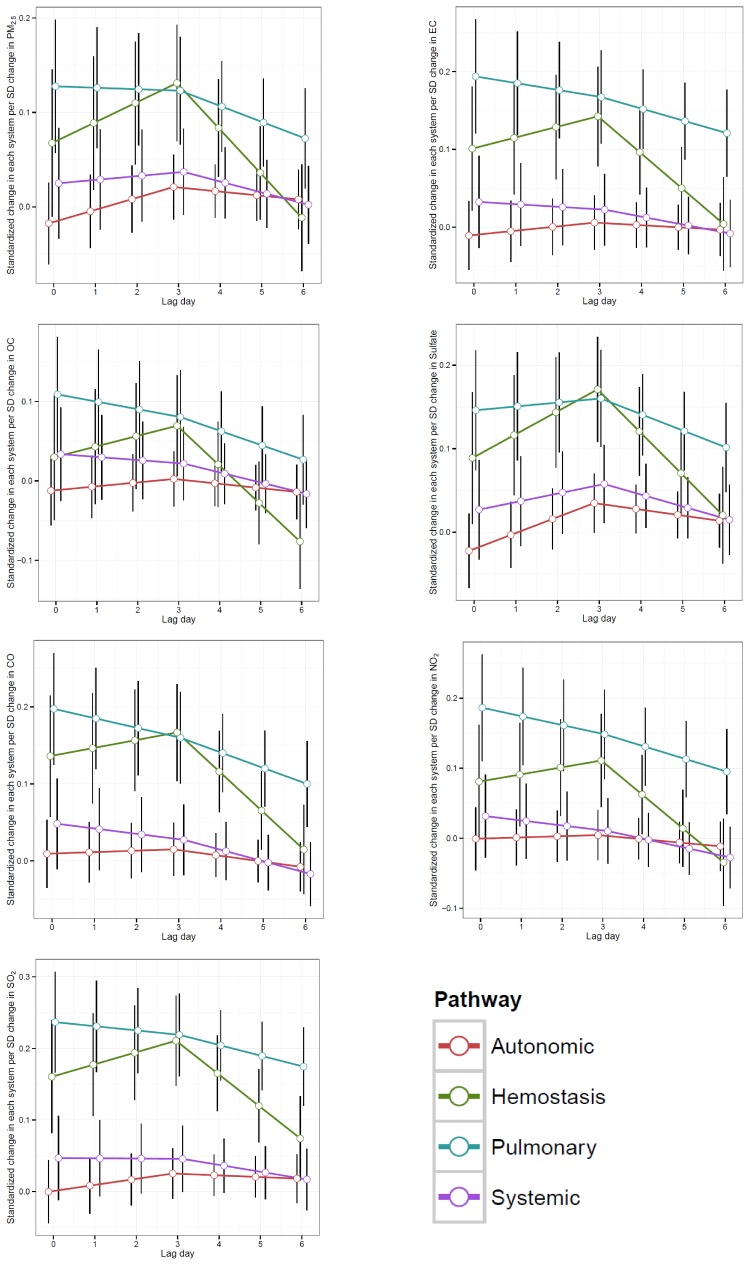
Mean association (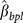) between standardized 24-hour average ambient air pollutant concentrations and the average (i.e., biomarker-level random effects are 0), standardized biomarker in each pathway from the Stage II model in Equation 2. Effect sizes are scaled to a 1 standard deviation change in each pollutant. The circle symbols represent the mean association coefficients and the black error bars represent their 95% confident intervals.

Fig. 3 shows predicted temporal patterns in 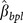 for the average biomarker in each of the four pathways, separately for each pollutant. These predictions were from the model that allowed temporal patterns to vary across both pathways and pollutants (Eq. 2). Variation in temporal patterns was evident across pathways but not across pollutants, except for sulfate. The most marked difference was that the pulmonary pathway associations with sulfate increased slightly until lag 3, whereas there was a consistent decrease in the pulmonary pathway associations with other pollutants across all lags. S1 Figure displays raw 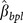 and biomarker-level predicted temporal patterns for associations with SO_2_, for the hemostasis pathway. To determine whether it was necessary to allow for differences in temporal patterns by pollutant, we conducted a likelihood ratio test comparing a model that excluded the 12 pollutant by lag interaction terms (terms with regression coefficients 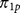 and 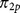 in Eq. 2) to the model that included these terms. We found evidence that at least one of the 12 regression coefficients was different from zero (p = 0.033 for the 12 *df* test). Since the pathway-specific temporal patterns appeared most different for sulfate, we created an indicator for sulfate (vs. all other pollutants) and compared the model with two pollutants by lag interactions (interactions between the sulfate indicator and each of the two lag variables) to the model with 12 pollutant by lag interaction terms (Eq. 2). The models were not significantly different (p = 0.27). Henceforth, we present results from this more parsimonious model. In this model, the estimated standard deviations of the biomarker-level random effects were largest for the random intercept (0.062 for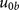) and smaller for the random slopes (0.0056 for 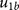
and 0.011 for 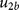).

### Results at lag 0

Candidate pathway groupings of biomarkers explained a significant amount of variation in 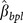 for each pollutant at lag 0 (p-values from likelihood ratio test with 3 df comparing models with and without the main effects of systems for: SO_2_: <0.0001, EC: 0.0002, CO: 0.0003, NO_2_: 0.0008, SO_4_^2−^: 0.0012, PM_2.5_: 0.0067, and OC: 0.044). Table 1 displays the mean 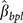 at lag 0 for the average biomarker in each pathway, by pollutant. For example, a 1-SD increase in SO_2_ at lag 0 (3.6 ppb) was associated with 0.166 (95% CI: 0.089, 0.244), 0.242 (95% CI: 0.173, 0.311), 0.053 (95% CI: −0.005, 0.110), and 0.006 (95% CI: −0.036, 0.048) SD unit changes in the average biomarker in the hemostasis, pulmonary, systemic, and autonomic pathways, respectively. At lag 0, there were strong increasing associations between the average biomarker in the pulmonary pathway and all seven pollutants and between the average biomarker in the hemostasis pathway and five pollutants (SO_2_, CO, EC, PM_2.5_, and SO_4_^2−^), while the confidence intervals of the associations with average biomarkers in the other two pathways included zero for all pollutants.

**Table 1:** Mean association (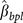) between standardized 24 hour average ambient air pollutant concentrations and the average (i.e., biomarker-level random effects are 0), standardized biomarker in each pathway, on the day of assessment (lag 0). Effect sizes are scaled to a 1 standard deviation change in each pollutant.

	Hemostasis		Pulmonary Inflammation & oxidative stress		Systemic Inflammation & oxidative stress		Autonomic function	
	Estimate	(95% CI)	Estimate	(95% CI)	Estimate	(95% CI)	Estimate	(95% CI)
CO	0.125	(0.047, 0.202)	0.185	(0.114, 0.257)	0.037	(−0.020, 0.094)	−0.003	(−0.045, 0.039)
EC	0.1	(0.022, 0.179)	0.193	(0.121, 0.265)	0.032	(−0.025, 0.090)	−0.011	(−0.053, 0.031)
NO_2_	0.071	(−0.009, 0.151)	0.176	(0.101, 0.251)	0.022	(−0.035, 0.080)	−0.011	(−0.053, 0.032)
OC	0.026	(−0.052, 0.104)	0.105	(0.034, 0.176)	0.03	(−0.027, 0.088)	−0.016	(−0.058, 0.026)
SO_2_	0.166	(0.089, 0.244)	0.242	(0.173, 0.311)	0.053	(−0.005, 0.110)	0.006	(−0.036, 0.048)
Sulfate	0.089	(0.010, 0.167)	0.145	(0.074, 0.217)	0.027	(−0.032, 0.086)	−0.022	(−0.066, 0.022)
PM_2.5_	0.082	(0.005, 0.158)	0.141	(0.072, 0.211)	0.039	(−0.018, 0.096)	−0.003	(−0.045, 0.038)

### Temporal patterns

Under the parsimonious model, the temporal patterns of the biomarker-pollutant-lag associations varied across candidate pathways (p<0.0001) and were not linear (from lag 0 to lag 3: p = 0.0629, from lag 3 to lag 6: p = 0.0005). Table 2 and Table 3 summarize pathway-specific temporal patterns for sulfate and for all other pollutants. For example, over a 7-day period the magnitude of the association between standardized pollutants and the average biomarker in the hemostasis pathway initially increased and later decreased. For sulfate, mean 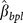 for the average biomarker in the hemostasis pathway increased by 0.028 (95% CI: 0.014, 0.041) SD units per day before lag 3 and decreased by 0.050 (95% CI: 0.028, 0.072) SD units per day after lag 3. Non-sulfate pollutants had a less steep initial rate of increase (mean 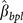 increased by 0.015 (95% CI: 0.004, 0.026) SD units per day before lag 3) and a similar rate of later decline by −0.048 (95% CI: −0.069, −0.026) SD units per day.

**Table 2:** For sulfate, rate of change per lag day in the mean association (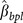) between standardized 24 hour average ambient sulfate concentrations and the average, standardized biomarker in each pathway.

	Before lag 3		After lag 3		
	Estimates*	(95% CI)	Estimates**	(95% CI)	P value***
Autonomic	0.019	(0.010, 0.028)	−0.007	(−0.019, 0.005)	0.004
Hemostasis	0.028	(0.014, 0.041)	−0.050	(−0.072, −0.028)	<0.0001
Pulmonary	0.005	(−0.008, 0.018)	−0.020	(−0.038, −0.001)	0.052
Systemic	0.01	(0.0002, 0.020)	−0.014	(−0.030, 0.002)	0.021

*Slope on first lag term, when the biomarker-level random effects are 0: 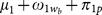
_._

**Slope on lag terms after lag 3, when the biomarker-level random effects are 0: 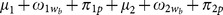

***p-value for a test of a difference in slope before and after lag 3.

**Table 3:** For pollutants other than sulfate, rate of change per lag day in the mean association (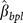) between standardized 24 hour average pollutant concentrations and the average, standardized biomarker in each pathway.

	Before lag 3		After lag 3		
	Estimates*	(95% CI)	Estimates**	(95% CI)	P value***
Autonomic	0.006	(0.001, 0.012)	−0.005	(−0.016, 0.007)	0.0568
Hemostasis	0.015	(0.004, 0.026)	−0.048	(−0.069, −0.026)	<0.0001
Pulmonary	−0.008	(−0.019, 0.004)	−0.017	(−0.035, 0.001)	0.3877
Systemic	−0.003	(−0.009, 0.004)	−0.012	(−0.027, 0.003)	0.1741

*Slope on first lag term, when the biomarker-level random effects are 0: 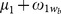

**Slope on lag terms after lag 3, when the biomarker-level random effects are 0: 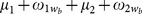
_._

***p-value for a test of a difference in slope before and after lag 3.

### Grouping biomarkers into candidate pathways

The parsimonious Stage II model included biomarker-level random effects (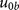, 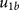, 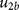) and fixed effects for the candidate pathways (ω_0*w*_, θ_0*wp*_ *,* ω_1*w*_, ω_2*wp*_) into which we had *a priori* grouped the biomarkers. By including the fixed-effects for pathways, we reduced the standard deviations of: (a) the biomarker-level random intercept (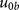) from 0.087 to 0.062, (b) the biomarker-level random slope on lag (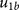) from 0.0078 to 0.0056, and (c) the biomarker-level random slope on lag 3 (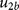) from 0.021 to 0.011, as compared to a model with no fixed-effects for pathways. As mentioned earlier, there was strong evidence for differences across pathways in 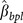 at lag 0 (p<0.05 for each pollutant) and over time (p<0.0001). Hence our *a priori* grouping of biomarkers into candidate pathways succeeded in explaining biomarker-level variation in 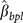. Since biomarkers such as fibrinogen and VWF could be categorized into the hemostasis and/or systemic inflammation and oxidative stress pathways, we conducted a sensitivity analysis (S2 Figure) in which we evaluated the impact of: (a) removing VWF from the hemostasis pathway and (b) moving fibrinogen from the systemic to the hemostasis pathway. Neither change altered the final interpretations of our analysis.

## Discussion

To the best of our knowledge this is the first study to systematically evaluate temporal patterns of associations between ambient air pollutants and candidate physiological pathways, using simultaneously collected biomarkers in an epidemiologic study. We confirmed that biomarkers grouped into candidate physiological pathways had some similarities in their associations with 7 pollutants over a 7 day period (Fig. 2). It appeared that the pulmonary inflammation and oxidative stress pathway was an early responder while the hemostasis pathway was a gradual responder. There was no consistent evidence in this study population for associations of the pollutants with the systemic or autonomic pathways. An explanation could be that we studied young adults without predisposing conditions thought to increase susceptibility to the autonomic effects of air pollution and considered only short exposure intervals [1].

In the following paragraphs, we present highlights of the relevant literature for each of our candidate physiological pathways. Many studies reported air pollution associations only with individual biomarkers, so in the discussion below we emphasize pathway-oriented studies and reviews.

### Pulmonary inflammation and oxidative stress

Ambient air pollution has been consistently associated with pulmonary markers of inflammation and oxidative stress, for example in Delfino et al. (2010) [9] and Laumbach and Kipen (2010) [10], but a recent review and meta-analysis reported significant heterogeneity across oxidative stress markers in blood, urine and airways and across studies [11].

### Autonomic function

A meta-analysis of 29 epidemiological studies indicates that PM_2.5_ was the only pollutant consistently associated with a decrease in HRV, with this association observed over several time-scales [12]. In the Normative Aging Study (NAS), PM_2.5_ and O_3_ were associated with decreased HRV over 4, 24 and 48 hour moving averages, but no associations were seen with NO_2_, SO_2_ and CO [13]. The time course of the effect of air pollution on HRV remains unclear. Studies have found associations within minutes [14], <2 hours [15], 4–6 hours [16], 1–2 days[13], as well as over a year [17].

### Hemostasis

In a previous study of 3256 people, plasma viscosity increased markedly during a 13 day air pollution exacerbation [18]. Similarly, fibrinogen has been positively associated with air pollution in several studies, though the time course of the response is not consistent [19], [20]. Global tests of coagulation, such as prothrombin time (PT), activated PT, and other coagulation proteins demonstrated less consistent associations. For example, in a large study (N = 1218) from the Lombardia Region in Italy, air pollution levels (PM_10_, CO, and NO_2_) in the hours preceding blood sampling were associated with shortened PT, but the relationship with activated PT, fibrinogen and the natural anticoagulant proteins was either null or protective [21]. Internal inconsistency amongst different biomarkers of hemostasis has been found elsewhere [22] and across the literature with VWF, fibrinogen, and platelet concentrations 26-Nov. 

Further, the role of air pollution on venous thromboembolism (VTE) remains inconsistent, with some studies indicating an increased risk among those who had higher air pollution exposure [29], [30]. However recent prospective studies do not support this association [31], [32].

### Systemic inflammation and oxidative stress

Studies report that PM is associated with increases in systemic inflammation as measured by CRP among elderly [33]. However, changes in CRP were not seen among the elderly in the NAS over multiple time frames of PM exposure, but changes in other inflammatory markers such as fibrinogen, ICAM and VCAM were observed. Other pollutants such as NO_2_, SO_4_^2−^ and O_3_ had different time patterns of associations with these markers [19].

This literature demonstrates the difficulties of drawing conclusions regarding the time course of air pollutant effects on candidate pathways using studies of individual biomarkers. Alternative approaches include a recent analysis of NAS data used structural equation modeling to estimate the mean effect of traffic-related air pollution on inflammation in the elderly by estimating a latent variable for inflammation from 3 biomarkers of inflammation and a latent variable for traffic-related air pollution [34], [35]. Our approach differs because we used fixed effects for multiple pathways rather than a latent variable for a single pathway, considered multiple measured ambient air pollutants, and investigated pathway-specific temporal patterns of association.

Strengths of this study include the unique design of “high-low-high” pollutant levels which led to large exposure contrasts among the pre-, the during-, and the post-Olympic periods (S1 Table) [36], repeated measurements on the same participants, simultaneous collection of a large number of biomarkers, and consideration of multiple lags of exposure. General limitations of this study have been discussed in detail previously [5], [6], [8]. In brief, there is possible non-differential exposure misclassification due to utilization of ambient air pollution levels rather than personal exposure assessment. There could be residual confounding in Stage I models, but the study protocol was designed to limit this possibility and several sensitivity analyses were performed.

Two assumptions of our approach merit discussion. First, in Stage I we fit 7 sets of single pollutant models rather than a single multi-pollutant model due to multi-collinearity of the 7 pollutants, and likewise fitted separate models for each lag rather than attempting a distributed-lag model in the first stage. Previous publications from our study have found that multi-pollutant models including 2 pollutants have similar, but attenuated associations as compared to single pollutant models [6], [7]. Our approach does not overcome the standard issues with correlated exposure metrics. We may have observed similar patterns in 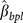 across pollutants at lag 0 (e.g. for the pulmonary pathway, all pollutants had relatively large positive 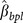) and over time (e.g. similar trajectories across all pollutants except SO_2_) due simply to the correlation of pollutants or due to the measured pollutant values being different surrogates for a latent true exposure. However, our mixed effects model aims to allow for these correlations by including random effects for pollutants and for biomarkers within pathways, and by the AR-1 correlation structure for lags. Also, we may have failed to find differences in trajectories across pollutants due to decreased power to detect higher-ordered interactions.

Second, the concept of “average biomarker” arises from the mixed effects model framework, with biomarker-random effects. This sort of interpretation allows for parsimonious way to describe the pathway-level effects. However, a limitation of this type of interpretation is that there may be no actual biomarker in the pathway with these effects. We grouped biomarkers into candidate pathways based on previous work [7]. Pathway-specific interpretations of our results assume that we have included biomarkers representative of the pathway. Selection of the biomarkers for this analysis was not based on the direction and statistical significance of the pollutant-biomarker association, which makes the interpretation of our results less subject to publication bias that often affects meta-analyses or literature reviews. Stage II results on pathway-level associations are essentially pathway-level averages of biomarker-specific associations. Biomarker-specific associations may vary within a pathway due to measurement error and sampling variation as well as from inherent differences in underlying biology. The Stage II mixed model accounts for biomarker-level variation within pathways using biomarker-level random effects and the variance of these random effects is assumed to be the same for each pathway. The Stage II model also assumes independence of the pathways. However, pathways could be physiologically interrelated or could overlap. Sensitivity analyses indicated that regrouping VWF or Fibrinogen did not alter our final conclusions.

Overall, our results suggest that among this healthy young adult population, the pulmonary inflammation and oxidative stress pathway is the first to respond to ambient air pollution exposure (within 24 hours) and the hemostasis pathway responds gradually over a 2–3 day period. The initial pulmonary response may contribute to the more gradual systemic changes that likely ultimately involve the cardiovascular system, such as hemostatic procoagulant responses and low grade systemic inflammation and oxidative stress pathways, or these responses may be independent. This sequence of events is consistent with other literature suggesting that pulmonary inflammation may drive systemic inflammatory response resulting in higher myocardial infarctions among COPD patients [37].

## Supporting Information

S1 Figure
**Raw** 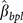 **and the biomarker-level predicted temporal patterns for associations with SO_2_, for the hemostasis pathway.**
(DOC)

S2 Figure **Sensitivity analyses evaluating the impact of: (a) removing vWF from the hemostasis pathway and (b) moving fibrinogen from the systemic to the hemostasis pathway.**
(DOC)

S1 Table **Air Pollution Statistics by Period Based on Time-series Models.**
(DOC)

S2 Table
**Description of pollutant-biomarker effects [Mean 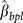 (SD)] from stage I models across pathways, pollutants and lag days.**
(DOC)

S3 Table **Estimated coefficients of pathways and the included biomarkers with sulfate at lag 0–6 by Stage II models.**
(DOC)

S4 Table **Estimated coefficients of pathways and the included biomarkers with CO at lag 0–6 by Stage II models.**
(DOC)

S5 Table **Estimated coefficients of pathways and the included biomarkers with SO_2_ at lag 0–6 by Stage II models.**
(DOC)

S6 Table **Estimated coefficients of pathways and the included biomarkers with NO_2_ at lag 0–6 by Stage II models.**
(DOC)

S7 Table **Estimated coefficients of pathways and the included biomarkers with PM_2.5_ at lag 0–6 by Stage II models.**
(DOC)

S8 Table **Estimated coefficients of pathways and the included biomarkers with elemental carbon at lag 0–6 by Stage II models.**
(DOC)

S9 Table **Estimated coefficients of pathways and the included biomarkers with organic carbon at lag 0–6 by Stage II models.**
(DOC)

S1 Appendix **Description of air pollution measurement.**
(DOC)

S2 Appendix **Description of biomarker measurement.**
(DOC)
