# Supplementary material for: The Cardiopulmonary Effects of Ambient Air Pollution and Mechanistic Pathways: A Comparative Hierarchical Pathway Analysis
Source: PLoS One. 2014 Dec 12;9(12):e114913. doi: 10.1371/journal.pone.0114913 (PMC4264846; doi:10.1371/journal.pone.0114913)
Supplement: S1 Figure — Raw and the biomarker-level predicted temporal patterns for associations with SO2, for the hemostasis pathway. (DOC) [file pone.0114913.s001.doc]

***Figure S1.*** Raw $\hat{\beta}_{bpl}$ and the biomarker-level predicted temporal patterns for associations with SO_2_, for the hemostasis pathway. The symbols represent the raw data from the stage I models, the dashed lines represent the biomarker-specific predicted data from stage II models, and the solid line represents the pathway-specific data from stage II models.
