# Supplementary material for: The Cardiopulmonary Effects of Ambient Air Pollution and Mechanistic Pathways: A Comparative Hierarchical Pathway Analysis
Source: PLoS One. 2014 Dec 12;9(12):e114913. doi: 10.1371/journal.pone.0114913 (PMC4264846; doi:10.1371/journal.pone.0114913)
Supplement: S2 Figure — Sensitivity analyses evaluating the impact of: (a) removing vWF from the hemostasis pathway and (b) moving fibrinogen from the systemic to the hemostasis pathway. (DOC) [file pone.0114913.s002.doc]

***Figure S2.*** Sensitivity analyses evaluating the impact of: (a) removing vWF from the hemostasis pathway and (b) moving fibrinogen from the systemic to the hemostasis pathway. Raw $\hat{\beta}_{bpl}$ and the biomarker-level predicted temporal patterns for associations with SO_2_, for the hemostasis pathway. The symbols represent the raw data from the stage I models, the dashed lines represent the biomarker-specific predicted data from stage II models, and the solid line represents the pathway-specific data from stage II models.

| (a) | (b) |
| --- | --- |
