# Supplementary material for: The Cardiopulmonary Effects of Ambient Air Pollution and Mechanistic Pathways: A Comparative Hierarchical Pathway Analysis
Source: PLoS One. 2014 Dec 12;9(12):e114913. doi: 10.1371/journal.pone.0114913 (PMC4264846; doi:10.1371/journal.pone.0114913)
Supplement: S1 Table — Air Pollution Statistics by Period Based on Time-series Models. (DOC) [file pone.0114913.s003.doc]

***Table S1.*** Air Pollution Statistics by Period Based on Time-series Models

| **Air Pollutant** | **Pre-Olympics** | | **During Olympics** | | **Post-Olympics** | | **Percent change (%)** | |
| --- | --- | --- | --- | --- | --- | --- | --- | --- |
|  | ***Mean*** | ***SE*** | ***Mean*** | ***SE*** | ***Mean*** | ***SE*** | ***Pre to During***  ***Mean (95% CI)*** | ***During to Post***  ***Mean (95% CI)*** |
| PM_2.5_ (μg/m^3^) | 98.9 | 14.7 | 71.9 | 15.1 | 85.3 | 15.3 | -27 (-64, 9) | 19 (-66, 103) |
| EC (μg/m^3^) | 2.2 | 0.3 | 1.4 | 0.3 | 3.4 | 0.3 | -36 (-70, -2) | 133 (35, 232) |
| OC (μg/m^3^) | 8.8 | 1.6 | 6.8 | 1.7 | 15.0 | 1.7 | -22 (-70, 25) | 120 (28, 211) |
| Sulfate (μg/m^3^) | 26.5 | 5.8 | 23.0 | 6.4 | 13.7 | 6.2 | -13 (-73, 47) | -41 (-133, 52) |
| SO_2_ (ppb) | 7.45 | 1.17 | 2.97 | 1.33 | 6.81 | 1.22 | -60 (-97, -23) | 129 (-224, 483) |
| CO (ppm) | 1.23 | 0.13 | 0.64 | 0.14 | 0.81 | 0.14 | -48 (-73, -24) | 27 (-114, 168) |
| NO_2_ (ppb) | 25.60 | 3.66 | 14.61 | 3.76 | 41.39 | 3.81 | -43 (-76, -10) | 183 (49, 317) |
