# Supplementary material for: The Cardiopulmonary Effects of Ambient Air Pollution and Mechanistic Pathways: A Comparative Hierarchical Pathway Analysis
Source: PLoS One. 2014 Dec 12;9(12):e114913. doi: 10.1371/journal.pone.0114913 (PMC4264846; doi:10.1371/journal.pone.0114913)
Supplement: S2 Table — Description of pollutant-biomarker effects [Mean (SD)] from stage I models across pathways, pollutants and lag days. (DOC) [file pone.0114913.s004.doc]

***Table S2.*** Description of pollutant-biomarker effects [Mean $\hat{\beta}_{bpl}$ (SD)] from stage I models across pathways, pollutants and lag days

_____________________________________________________________________________________________________

Lag=0 Lag=1 Lag=2 Lag=3 Lag=4 Lag=5 Lag=6

_____________________________________________________________________________________________________

*Autonomic (N=10)*

CO 0.01 (0.04) 0.00 (0.05) 0.01 (0.04) 0.02 (0.05) 0.02 (0.02) 0.00 (0.03) -0.01 (0.05)

EC -0.01 (0.05) -0.01 (0.05) -0.01 (0.04) 0.01 (0.05) 0.01 (0.02) 0.00 (0.05) 0.00 (0.06)

NO2 -0.01 (0.07) 0.00 (0.06) 0.00 (0.04) 0.01 (0.07) 0.01 (0.04) -0.01 (0.05) -0.01 (0.06)

OC -0.01 (0.06) -0.01(0.05) -0.02 (0.04) 0.01 (0.06) 0.01 (0.02) -0.01 (0.04) -0.01 (0.07)

SO2 0.01 (0.03) 0.01 (0.05) 0.01 (0.04) 0.02 (0.04) 0.03 (0.02) 0.01 (0.02) 0.01 (0.04)

Sulfate -0.01 (0.03) 0.00 (0.05) 0.01 (0.04) 0.04 (0.02) 0.05 (0.05) 0.02 (0.05) 0.01 (0.05)

PM2.5 -0.01 (0.04) 0.00 (0.04) -0.01 (0.04) 0.03 (0.03) 0.03 (0.04) 0.01 (0.05) 0.00 (0.04)

*Hemostasis (N=3)*

CO 0.10 (0.12) 0.16 (0.16) 0.16 (0.14) 0.16 (0.11) 0.14 (0.10) 0.05 (0.09) 0.03 (0.12)

EC 0.11 (0.13) 0.10 (0.09) 0.12 (0.14) 0.15 (0.08) 0.09 (0.07) 0.01 (0.10) 0.02 (0.04)

NO2 0.09 (0.11) 0.13 (0.13) 0.08 (0.12) 0.12 (0.09) 0.08 (0.10) 0.00 (0.10) -0.02 (0.15)

OC 0.04 (0.08) 0.07 (0.07) 0.03 (0.11) 0.08 (0.07) 0.07 (0.07) -0.07 (0.08) -0.09 (0.02)

SO2 0.14 (0.10) 0.17 (0.12) 0.24 (0.22) 0.20 (0.14) 0.16 (0.07) 0.13 (0.05) 0.12 (0.13)

Sulfate 0.09 (0.10) 0.11 (0.10) 0.16 (0.18) 0.20 (0.07) 0.14 (0.02) 0.02 (0.11) 0.02 (0.04)

PM2.5 0.08 (0.11) 0.08 (0.09) 0.11 (0.15) 0.15 (0.08) 0.11 (0.06) 0.00 (0.05) -0.01 (0.03)

*Pulmonary inflammation and oxidative stress (N=4)*

CO 0.23 (0.14) 0.17 (0.11) 0.21 (0.09) 0.18 (0.06) 0.15 (0.06) 0.12 (0.08) 0.00 (0.10)

EC 0.26 (0.11) 0.13 (0.05) 0.07 (0.08) 0.15 (0.06) 0.14 (0.07) 0.18 (0.06) 0.14 (0.07)

NO2 0.21 (0.14) 0.18 (0.10) 0.11 (0.08) 0.14 (0.08) 0.12 (0.03) 0.17 (0.02) 0.08 (0.07)

OC 0.14 (0.10) 0.08 (0.08) 0.02 (0.05) 0.07 (0.07) 0.11 (0.03) 0.08 (0.08) -0.04 (0.07)

SO2 0.26 (0.06) 0.18 (0.08) 0.24 (0.13) 0.25 (0.07) 0.21 (0.11) 0.18 (0.09) 0.16 (0.14)

Sulfate 0.16 (0.12) 0.08 (0.12) 0.11 (0.12) 0.18 (0.14) 0.19 (0.13) 0.15 (0.14) 0.11 (0.17)

PM2.5 0.17 (0.12) 0.07 (0.09) 0.08 (0.07) 0.14 (0.08) 0.12 (0.09) 0.10 (0.09) 0.07 (0.15)

*Systemic inflammation and oxidative stress (N=5)*

CO 0.04 (0.05) 0.06 (0.07) 0.03 (0.07) 0.02 (0.08) 0.00 (0.06) 0.01 (0.08) 0.02 (0.10)

EC 0.04 (0.06) 0.04 (0.10) 0.05 (0.08) 0.04 (0.07) -0.01 (0.04) -0.01 (0.06) 0.00 (0.02)

NO2 0.04 (0.07) 0.06 (0.11) 0.04 (0.10) 0.03 (0.10) 0.01 (0.08) 0.01 (0.10) 0.02 (0.10)

OC 0.03 (0.05) 0.04 (0.08) 0.03 (0.07) 0.03 (0.07) 0.00 (0.05) -0.03 (0.05) 0.00 (0.05)

SO2 0.02 (0.04) 0.04 (0.07) 0.04 (0.07) 0.04 (0.04) 0.02 (0.06) 0.01 (0.07) 0.04 (0.10)

Sulfate 0.03 (0.08) 0.04 (0.08) 0.06 (0.07) 0.04 (0.04) 0.00 (0.03) -0.01 (0.05) -0.01 (0.03)

PM2.5 0.03 (0.08) 0.04 (0.08) 0.05 (0.08) 0.04 (0.06) 0.00 (0.02) -0.02 (0.03) 0.00 (0.02)

_____________________________________________________________________________________________________
