# Supplementary material for: The Cardiopulmonary Effects of Ambient Air Pollution and Mechanistic Pathways: A Comparative Hierarchical Pathway Analysis
Source: PLoS One. 2014 Dec 12;9(12):e114913. doi: 10.1371/journal.pone.0114913 (PMC4264846; doi:10.1371/journal.pone.0114913)
Supplement: S3 Table — Estimated coefficients of pathways and the included biomarkers with sulfate at lag 0–6 by Stage II models. (DOC) [file pone.0114913.s005.doc]

***Table S3.*** Estimated coefficients of pathways and the included biomarkers with sulfate at lag 0-6 by Stage II models.

| Pathway and biomarker | Lag=0 | Lag=1 | Lag=2 | Lag=3 | Lag=4 | Lag=5 | Lag=6 |
| --- | --- | --- | --- | --- | --- | --- | --- |
| **Autonomic function** | **-0.022** | **-0.003** | **0.016** | **0.035** | **0.028** | **0.021** | **0.014** |
| DBP | -0.021 | -0.004 | 0.013 | 0.030 | 0.011 | -0.008 | -0.027 |
| SBP | 0.016 | 0.031 | 0.047 | 0.063 | 0.045 | 0.027 | 0.009 |
| Heart Rate | 0.007 | 0.024 | 0.041 | 0.057 | 0.044 | 0.031 | 0.018 |
| HF | -0.062 | -0.037 | -0.013 | 0.012 | 0.025 | 0.038 | 0.050 |
| LF | -0.013 | 0.004 | 0.020 | 0.037 | 0.020 | 0.004 | -0.013 |
| LF/HF | 0.019 | 0.031 | 0.043 | 0.055 | 0.025 | -0.004 | -0.034 |
| rMSSD | -0.080 | -0.054 | -0.029 | -0.004 | 0.009 | 0.021 | 0.033 |
| SDNN | -0.068 | -0.043 | -0.019 | 0.006 | 0.017 | 0.027 | 0.038 |
| VLF | 0.011 | 0.029 | 0.047 | 0.066 | 0.060 | 0.055 | 0.050 |
| Total power | -0.032 | -0.013 | 0.007 | 0.027 | 0.022 | 0.018 | 0.013 |
| **Hemostasis** | **0.089** | **0.116** | **0.144** | **0.171** | **0.121** | **0.071** | **0.021** |
| sCD62P | 0.205 | 0.223 | 0.242 | 0.260 | 0.186 | 0.113 | 0.039 |
| sCD40L | -0.016 | 0.021 | 0.059 | 0.096 | 0.072 | 0.049 | 0.025 |
| VWF | 0.077 | 0.104 | 0.131 | 0.157 | 0.104 | 0.051 | -0.002 |
| **Pulmonary inflammation and oxidative stress** | **0.146** | **0.151** | **0.155** | **0.160** | **0.141** | **0.121** | **0.102** |
| EBC nitrite | 0.148 | 0.149 | 0.150 | 0.151 | 0.114 | 0.077 | 0.041 |
| FeNO | 0.219 | 0.221 | 0.223 | 0.226 | 0.210 | 0.195 | 0.179 |
| EBC pH | 0.138 | 0.142 | 0.146 | 0.150 | 0.128 | 0.106 | 0.083 |
| MDA | 0.080 | 0.091 | 0.103 | 0.114 | 0.110 | 0.107 | 0.103 |
| **Systemic inflammation and oxidative stress** | **0.027** | **0.037** | **0.048** | **0.058** | **0.043** | **0.029** | **0.015** |
| Urinary 8-OHdG | 0.129 | 0.132 | 0.135 | 0.138 | 0.109 | 0.079 | 0.049 |
| Fibrinogen | 0.022 | 0.033 | 0.043 | 0.054 | 0.042 | 0.031 | 0.019 |
| WBC | -0.029 | -0.017 | -0.005 | 0.007 | -0.005 | -0.018 | -0.031 |
| RBC | -0.053 | -0.037 | -0.020 | -0.004 | -0.007 | -0.011 | -0.014 |
| Urinary MDA | 0.067 | 0.075 | 0.084 | 0.092 | 0.079 | 0.065 | 0.051 |
