# Supplementary material for: The Cardiopulmonary Effects of Ambient Air Pollution and Mechanistic Pathways: A Comparative Hierarchical Pathway Analysis
Source: PLoS One. 2014 Dec 12;9(12):e114913. doi: 10.1371/journal.pone.0114913 (PMC4264846; doi:10.1371/journal.pone.0114913)
Supplement: S4 Table — Estimated coefficients of pathways and the included biomarkers with CO at lag 0–6 by Stage II models. (DOC) [file pone.0114913.s006.doc]

***Table S4.*** Estimated coefficients of pathways and the included biomarkers with CO at lag 0-6 by Stage II models.

| Pathway and biomarker | Lag=0 | Lag=1 | Lag=2 | Lag=3 | Lag=4 | Lag=5 | Lag=6 |
| --- | --- | --- | --- | --- | --- | --- | --- |
| **Autonomic function** | **0.009** | **0.011** | **0.013** | **0.015** | **0.007** | **0.000** | **-0.008** |
| DBP | 0.011 | 0.011 | 0.010 | 0.010 | -0.009 | -0.029 | -0.048 |
| SBP | 0.047 | 0.046 | 0.044 | 0.043 | 0.024 | 0.006 | -0.013 |
| Heart Rate | 0.039 | 0.038 | 0.038 | 0.037 | 0.024 | 0.011 | -0.003 |
| HF | -0.031 | -0.023 | -0.015 | -0.008 | 0.004 | 0.017 | 0.029 |
| LF | 0.019 | 0.018 | 0.017 | 0.017 | 0.000 | -0.017 | -0.034 |
| LF/HF | 0.051 | 0.046 | 0.041 | 0.036 | 0.005 | -0.025 | -0.056 |
| rMSSD | -0.048 | -0.040 | -0.032 | -0.024 | -0.012 | 0.000 | 0.012 |
| SDNN | -0.036 | -0.029 | -0.021 | -0.014 | -0.004 | 0.007 | 0.017 |
| VLF | 0.043 | 0.044 | 0.045 | 0.046 | 0.040 | 0.034 | 0.028 |
| Total power | -0.001 | 0.002 | 0.004 | 0.007 | 0.002 | -0.003 | -0.008 |
| **Hemostasis** | **0.136** | **0.146** | **0.157** | **0.167** | **0.116** | **0.065** | **0.015** |
| sCD62P | 0.252 | 0.254 | 0.255 | 0.256 | 0.182 | 0.107 | 0.033 |
| sCD40L | 0.031 | 0.052 | 0.072 | 0.092 | 0.068 | 0.043 | 0.019 |
| VWF | 0.125 | 0.134 | 0.143 | 0.153 | 0.099 | 0.046 | -0.008 |
| **Pulmonary inflammation and oxidative stress** | **0.197** | **0.185** | **0.172** | **0.160** | **0.140** | **0.120** | **0.100** |
| EBC nitrite | 0.199 | 0.183 | 0.167 | 0.150 | 0.113 | 0.076 | 0.039 |
| FeNO | 0.270 | 0.255 | 0.240 | 0.225 | 0.209 | 0.193 | 0.177 |
| EBC pH | 0.189 | 0.176 | 0.163 | 0.150 | 0.127 | 0.104 | 0.082 |
| MDA | 0.132 | 0.126 | 0.120 | 0.114 | 0.110 | 0.106 | 0.102 |
| **Systemic inflammation and oxidative stress** | **0.048** | **0.041** | **0.034** | **0.027** | **0.013** | **-0.002** | **-0.017** |
| Urinary 8-OHdG | 0.150 | 0.136 | 0.122 | 0.108 | 0.078 | 0.048 | 0.018 |
| Fibrinogen | 0.043 | 0.037 | 0.030 | 0.024 | 0.012 | -0.001 | -0.013 |
| WBC | -0.007 | -0.013 | -0.018 | -0.023 | -0.036 | -0.050 | -0.063 |
| RBC | -0.032 | -0.033 | -0.034 | -0.034 | -0.038 | -0.042 | -0.046 |
| Urinary MDA | 0.088 | 0.079 | 0.071 | 0.062 | 0.048 | 0.034 | 0.020 |
