# Supplementary material for: The Cardiopulmonary Effects of Ambient Air Pollution and Mechanistic Pathways: A Comparative Hierarchical Pathway Analysis
Source: PLoS One. 2014 Dec 12;9(12):e114913. doi: 10.1371/journal.pone.0114913 (PMC4264846; doi:10.1371/journal.pone.0114913)
Supplement: S5 Table — Estimated coefficients of pathways and the included biomarkers with SO2 at lag 0–6 by Stage II models. (DOC) [file pone.0114913.s007.doc]

***Table S5.*** Estimated coefficients of pathways and the included biomarkers with SO_2_ at lag 0-6 by Stage II models.

| Pathway and biomarker | Lag=0 | Lag=1 | Lag=2 | Lag=3 | Lag=4 | Lag=5 | Lag=6 |
| --- | --- | --- | --- | --- | --- | --- | --- |
| **Autonomic function** | **0.000** | **0.008** | **0.017** | **0.025** | **0.023** | **0.020** | **0.018** |
| DBP | 0.001 | 0.008 | 0.014 | 0.020 | 0.006 | -0.008 | -0.023 |
| SBP | 0.038 | 0.043 | 0.048 | 0.053 | 0.040 | 0.026 | 0.013 |
| Heart Rate | 0.029 | 0.035 | 0.041 | 0.048 | 0.039 | 0.031 | 0.023 |
| HF | -0.040 | -0.026 | -0.012 | 0.002 | 0.020 | 0.037 | 0.055 |
| LF | 0.009 | 0.015 | 0.021 | 0.027 | 0.015 | 0.003 | -0.008 |
| LF/HF | 0.041 | 0.043 | 0.044 | 0.046 | 0.020 | -0.005 | -0.030 |
| rMSSD | -0.058 | -0.043 | -0.028 | -0.014 | 0.004 | 0.021 | 0.038 |
| SDNN | -0.046 | -0.032 | -0.018 | -0.004 | 0.012 | 0.027 | 0.042 |
| VLF | 0.033 | 0.041 | 0.048 | 0.056 | 0.055 | 0.055 | 0.054 |
| Total power | -0.010 | -0.001 | 0.008 | 0.017 | 0.017 | 0.017 | 0.017 |
| **Hemostasis** | **0.160** | **0.177** | **0.194** | **0.211** | **0.165** | **0.120** | **0.074** |
| sCD62P | 0.277 | 0.284 | 0.292 | 0.300 | 0.231 | 0.162 | 0.093 |
| sCD40L | 0.056 | 0.082 | 0.109 | 0.136 | 0.117 | 0.098 | 0.079 |
| VWF | 0.149 | 0.165 | 0.181 | 0.197 | 0.148 | 0.100 | 0.052 |
| **Pulmonary inflammation and oxidative stress** | **0.237** | **0.231** | **0.225** | **0.219** | **0.204** | **0.189** | **0.175** |
| EBC nitrite | 0.239 | 0.229 | 0.219 | 0.210 | 0.178 | 0.146 | 0.114 |
| FeNO | 0.309 | 0.301 | 0.293 | 0.285 | 0.274 | 0.263 | 0.252 |
| EBC pH | 0.228 | 0.222 | 0.216 | 0.209 | 0.192 | 0.174 | 0.156 |
| MDA | 0.171 | 0.172 | 0.172 | 0.173 | 0.174 | 0.175 | 0.176 |
| **Systemic inflammation and oxidative stress** | **0.047** | **0.046** | **0.046** | **0.046** | **0.036** | **0.026** | **0.017** |
| Urinary 8-OHdG | 0.148 | 0.141 | 0.134 | 0.126 | 0.101 | 0.076 | 0.051 |
| Fibrinogen | 0.041 | 0.042 | 0.042 | 0.042 | 0.035 | 0.028 | 0.021 |
| WBC | -0.009 | -0.007 | -0.006 | -0.005 | -0.013 | -0.021 | -0.029 |
| RBC | -0.033 | -0.028 | -0.022 | -0.016 | -0.015 | -0.013 | -0.012 |
| Urinary MDA | 0.086 | 0.084 | 0.082 | 0.080 | 0.071 | 0.062 | 0.053 |
