# Supplementary material for: The Cardiopulmonary Effects of Ambient Air Pollution and Mechanistic Pathways: A Comparative Hierarchical Pathway Analysis
Source: PLoS One. 2014 Dec 12;9(12):e114913. doi: 10.1371/journal.pone.0114913 (PMC4264846; doi:10.1371/journal.pone.0114913)
Supplement: S6 Table — Estimated coefficients of pathways and the included biomarkers with NO2 at lag 0–6 by Stage II models. (DOC) [file pone.0114913.s008.doc]

***Table S6.*** Estimated coefficients of pathways and the included biomarkers with NO_2_ at lag 0-6 by Stage II models.

| Pathway and biomarker | Lag=0 | Lag=1 | Lag=2 | Lag=3 | Lag=4 | Lag=5 | Lag=6 |
| --- | --- | --- | --- | --- | --- | --- | --- |
| **Autonomic function** | **-0.001** | **0.001** | **0.003** | **0.004** | **-0.001** | **-0.006** | **-0.012** |
| DBP | 0.001 | 0.000 | 0.000 | 0.000 | -0.018 | -0.035 | -0.052 |
| SBP | 0.037 | 0.036 | 0.034 | 0.032 | 0.016 | 0.000 | -0.017 |
| Heart Rate | 0.029 | 0.028 | 0.028 | 0.027 | 0.016 | 0.004 | -0.007 |
| HF | -0.041 | -0.033 | -0.026 | -0.018 | -0.004 | 0.011 | 0.025 |
| LF | 0.009 | 0.008 | 0.007 | 0.006 | -0.008 | -0.023 | -0.038 |
| LF/HF | 0.040 | 0.035 | 0.030 | 0.025 | -0.003 | -0.032 | -0.060 |
| rMSSD | -0.058 | -0.050 | -0.042 | -0.034 | -0.020 | -0.006 | 0.008 |
| SDNN | -0.046 | -0.039 | -0.032 | -0.025 | -0.012 | 0.000 | 0.013 |
| VLF | 0.033 | 0.033 | 0.034 | 0.035 | 0.031 | 0.028 | 0.024 |
| Total power | -0.011 | -0.008 | -0.006 | -0.003 | -0.006 | -0.009 | -0.012 |
| **Hemostasis** | **0.081** | **0.091** | **0.101** | **0.111** | **0.063** | **0.014** | **-0.034** |
| sCD62P | 0.197 | 0.198 | 0.199 | 0.200 | 0.128 | 0.056 | -0.016 |
| sCD40L | -0.024 | -0.004 | 0.016 | 0.036 | 0.014 | -0.008 | -0.030 |
| VWF | 0.069 | 0.079 | 0.088 | 0.097 | 0.046 | -0.006 | -0.057 |
| **Pulmonary inflammation and oxidative stress** | **0.186** | **0.174** | **0.161** | **0.148** | **0.131** | **0.113** | **0.095** |
| EBC nitrite | 0.188 | 0.172 | 0.155 | 0.139 | 0.104 | 0.069 | 0.034 |
| FeNO | 0.259 | 0.244 | 0.229 | 0.214 | 0.200 | 0.186 | 0.173 |
| EBC pH | 0.178 | 0.165 | 0.152 | 0.139 | 0.118 | 0.097 | 0.077 |
| MDA | 0.121 | 0.114 | 0.108 | 0.102 | 0.100 | 0.099 | 0.097 |
| **Systemic inflammation and oxidative stress** | **0.032** | **0.024** | **0.017** | **0.010** | **-0.002** | **-0.015** | **-0.027** |
| Urinary 8-OHdG | 0.133 | 0.119 | 0.105 | 0.091 | 0.063 | 0.035 | 0.007 |
| Fibrinogen | 0.026 | 0.020 | 0.013 | 0.007 | -0.003 | -0.014 | -0.024 |
| WBC | -0.024 | -0.029 | -0.035 | -0.040 | -0.051 | -0.062 | -0.074 |
| RBC | -0.048 | -0.049 | -0.051 | -0.052 | -0.053 | -0.055 | -0.056 |
| Urinary MDA | 0.071 | 0.062 | 0.054 | 0.045 | 0.033 | 0.021 | 0.009 |
