# Supplementary material for: The Cardiopulmonary Effects of Ambient Air Pollution and Mechanistic Pathways: A Comparative Hierarchical Pathway Analysis
Source: PLoS One. 2014 Dec 12;9(12):e114913. doi: 10.1371/journal.pone.0114913 (PMC4264846; doi:10.1371/journal.pone.0114913)
Supplement: S7 Table — Estimated coefficients of pathways and the included biomarkers with PM2.5 at lag 0–6 by Stage II models. (DOC) [file pone.0114913.s009.doc]

***Table S7. Estimated coefficients of pathways and the included biomarkers with PM_2.5_ at lag 0-6 by Stage II models.***

| Pathway and biomarker | Lag=0 | Lag=1 | Lag=2 | Lag=3 | Lag=4 | Lag=5 | Lag=6 |
| --- | --- | --- | --- | --- | --- | --- | --- |
| **Autonomic function** | **-0.018** | **-0.005** | **0.008** | **0.021** | **0.017** | **0.012** | **0.008** |
| DBP | -0.016 | -0.005 | 0.006 | 0.016 | 0.000 | -0.017 | -0.033 |
| SBP | 0.021 | 0.030 | 0.039 | 0.049 | 0.033 | 0.018 | 0.003 |
| Heart Rate | 0.012 | 0.022 | 0.033 | 0.043 | 0.033 | 0.023 | 0.013 |
| HF | -0.057 | -0.039 | -0.020 | -0.002 | 0.014 | 0.029 | 0.044 |
| LF | -0.008 | 0.002 | 0.012 | 0.023 | 0.009 | -0.005 | -0.018 |
| LF/HF | 0.024 | 0.030 | 0.036 | 0.042 | 0.014 | -0.013 | -0.040 |
| rMSSD | -0.075 | -0.056 | -0.037 | -0.018 | -0.003 | 0.012 | 0.027 |
| SDNN | -0.063 | -0.045 | -0.026 | -0.008 | 0.005 | 0.019 | 0.032 |
| VLF | 0.016 | 0.028 | 0.040 | 0.052 | 0.049 | 0.046 | 0.044 |
| Total power | -0.028 | -0.014 | 0.000 | 0.013 | 0.011 | 0.009 | 0.007 |
| **Hemostasis** | **0.068** | **0.089** | **0.110** | **0.131** | **0.084** | **0.036** | **-0.011** |
| sCD62P | 0.184 | 0.196 | 0.208 | 0.220 | 0.149 | 0.078 | 0.007 |
| sCD40L | -0.037 | -0.006 | 0.025 | 0.056 | 0.035 | 0.014 | -0.007 |
| VWF | 0.056 | 0.077 | 0.097 | 0.117 | 0.067 | 0.016 | -0.034 |
| **Pulmonary inflammation and oxidative stress** | **0.128** | **0.126** | **0.125** | **0.123** | **0.106** | **0.089** | **0.072** |
| EBC nitrite | 0.129 | 0.124 | 0.119 | 0.114 | 0.080 | 0.046 | 0.012 |
| FeNO | 0.200 | 0.196 | 0.192 | 0.189 | 0.176 | 0.163 | 0.150 |
| EBC pH | 0.119 | 0.117 | 0.115 | 0.113 | 0.093 | 0.074 | 0.054 |
| MDA | 0.062 | 0.067 | 0.072 | 0.077 | 0.076 | 0.075 | 0.074 |
| **Systemic inflammation and oxidative stress** | **0.025** | **0.029** | **0.033** | **0.037** | **0.025** | **0.014** | **0.002** |
| Urinary 8-OHdG | 0.127 | 0.124 | 0.121 | 0.118 | 0.091 | 0.064 | 0.037 |
| Fibrinogen | 0.020 | 0.024 | 0.029 | 0.034 | 0.024 | 0.015 | 0.006 |
| WBC | -0.031 | -0.025 | -0.019 | -0.013 | -0.023 | -0.034 | -0.044 |
| RBC | -0.055 | -0.045 | -0.035 | -0.025 | -0.025 | -0.026 | -0.026 |
| Urinary MDA | 0.065 | 0.067 | 0.069 | 0.072 | 0.061 | 0.050 | 0.039 |
