# Supplementary material for: The Cardiopulmonary Effects of Ambient Air Pollution and Mechanistic Pathways: A Comparative Hierarchical Pathway Analysis
Source: PLoS One. 2014 Dec 12;9(12):e114913. doi: 10.1371/journal.pone.0114913 (PMC4264846; doi:10.1371/journal.pone.0114913)
Supplement: S8 Table — Estimated coefficients of pathways and the included biomarkers with elemental carbon at lag 0–6 by Stage II models. (DOC) [file pone.0114913.s010.doc]

***Table S8.*** Estimated coefficients of pathways and the included biomarkers with elemental carbon at lag 0-6 by Stage II models.

| Pathway and biomarker | Lag=0 | Lag=1 | Lag=2 | Lag=3 | Lag=4 | Lag=5 | Lag=6 |
| --- | --- | --- | --- | --- | --- | --- | --- |
| **Autonomic function** | **-0.011** | **-0.005** | **0.000** | **0.006** | **0.003** | **0.000** | **-0.003** |
| DBP | -0.009 | -0.006 | -0.002 | 0.001 | -0.014 | -0.029 | -0.044 |
| SBP | 0.027 | 0.029 | 0.031 | 0.034 | 0.020 | 0.006 | -0.008 |
| Heart Rate | 0.019 | 0.022 | 0.025 | 0.028 | 0.019 | 0.010 | 0.002 |
| HF | -0.051 | -0.039 | -0.028 | -0.017 | 0.000 | 0.017 | 0.033 |
| LF | -0.001 | 0.002 | 0.005 | 0.008 | -0.005 | -0.017 | -0.029 |
| LF/HF | 0.031 | 0.029 | 0.028 | 0.026 | 0.000 | -0.025 | -0.051 |
| rMSSD | -0.068 | -0.056 | -0.045 | -0.033 | -0.016 | 0.000 | 0.016 |
| SDNN | -0.056 | -0.045 | -0.034 | -0.023 | -0.008 | 0.006 | 0.021 |
| VLF | 0.023 | 0.027 | 0.032 | 0.036 | 0.035 | 0.034 | 0.033 |
| Total power | -0.021 | -0.015 | -0.008 | -0.002 | -0.003 | -0.003 | -0.004 |
| **Hemostasis** | **0.101** | **0.115** | **0.129** | **0.143** | **0.096** | **0.050** | **0.004** |
| sCD62P | 0.217 | 0.222 | 0.227 | 0.231 | 0.162 | 0.092 | 0.022 |
| sCD40L | -0.004 | 0.020 | 0.044 | 0.068 | 0.048 | 0.028 | 0.009 |
| VWF | 0.090 | 0.103 | 0.116 | 0.129 | 0.079 | 0.030 | -0.019 |
| **Pulmonary inflammation and oxidative stress** | **0.194** | **0.185** | **0.176** | **0.167** | **0.152** | **0.136** | **0.121** |
| EBC nitrite | 0.196 | 0.183 | 0.170 | 0.158 | 0.125 | 0.093 | 0.060 |
| FeNO | 0.266 | 0.255 | 0.244 | 0.233 | 0.221 | 0.210 | 0.198 |
| EBC pH | 0.185 | 0.176 | 0.167 | 0.157 | 0.139 | 0.121 | 0.103 |
| MDA | 0.128 | 0.126 | 0.123 | 0.121 | 0.122 | 0.122 | 0.123 |
| **Systemic inflammation and oxidative stress** | **0.032** | **0.029** | **0.026** | **0.023** | **0.012** | **0.002** | **-0.008** |
| Urinary 8-OHdG | 0.134 | 0.124 | 0.113 | 0.103 | 0.078 | 0.052 | 0.026 |
| Fibrinogen | 0.027 | 0.024 | 0.022 | 0.019 | 0.011 | 0.003 | -0.004 |
| WBC | -0.023 | -0.025 | -0.026 | -0.028 | -0.037 | -0.045 | -0.054 |
| RBC | -0.048 | -0.045 | -0.042 | -0.039 | -0.038 | -0.038 | -0.037 |
| Urinary MDA | 0.072 | 0.067 | 0.062 | 0.057 | 0.048 | 0.038 | 0.028 |
