# Supplementary material for: The Cardiopulmonary Effects of Ambient Air Pollution and Mechanistic Pathways: A Comparative Hierarchical Pathway Analysis
Source: PLoS One. 2014 Dec 12;9(12):e114913. doi: 10.1371/journal.pone.0114913 (PMC4264846; doi:10.1371/journal.pone.0114913)
Supplement: S9 Table — Estimated coefficients of pathways and the included biomarkers with organic carbon at lag 0–6 by Stage II models. (DOC) [file pone.0114913.s011.doc]

***Table S9.*** Estimated coefficients of pathways and the included biomarkers with organic carbon at lag 0-6 by Stage II models.

| Pathway and biomarker | Lag=0 | Lag=1 | Lag=2 | Lag=3 | Lag=4 | Lag=5 | Lag=6 |
| --- | --- | --- | --- | --- | --- | --- | --- |
| **Autonomic function** | **-0.012** | **-0.007** | **-0.003** | **0.002** | **-0.003** | **-0.008** | **-0.014** |
| DBP | -0.011 | -0.008 | -0.005 | -0.002 | -0.020 | -0.037 | -0.055 |
| SBP | 0.026 | 0.027 | 0.029 | 0.030 | 0.014 | -0.003 | -0.019 |
| Heart Rate | 0.017 | 0.020 | 0.022 | 0.025 | 0.013 | 0.002 | -0.009 |
| HF | -0.052 | -0.042 | -0.031 | -0.020 | -0.006 | 0.008 | 0.023 |
| LF | -0.003 | -0.001 | 0.002 | 0.004 | -0.011 | -0.025 | -0.040 |
| LF/HF | 0.029 | 0.027 | 0.025 | 0.023 | -0.005 | -0.034 | -0.062 |
| rMSSD | -0.070 | -0.059 | -0.047 | -0.036 | -0.022 | -0.008 | 0.006 |
| SDNN | -0.058 | -0.047 | -0.037 | -0.027 | -0.014 | -0.002 | 0.010 |
| VLF | 0.021 | 0.025 | 0.029 | 0.033 | 0.029 | 0.026 | 0.022 |
| Total power | -0.022 | -0.017 | -0.011 | -0.006 | -0.009 | -0.012 | -0.015 |
| **Hemostasis** | **0.030** | **0.043** | **0.056** | **0.069** | **0.021** | **-0.028** | **-0.076** |
| sCD62P | 0.146 | 0.150 | 0.154 | 0.158 | 0.086 | 0.014 | -0.058 |
| sCD40L | -0.075 | -0.052 | -0.029 | -0.005 | -0.028 | -0.050 | -0.072 |
| VWF | 0.018 | 0.031 | 0.043 | 0.056 | 0.004 | -0.048 | -0.099 |
| **Pulmonary inflammation and oxidative stress** | **0.109** | **0.099** | **0.090** | **0.080** | **0.062** | **0.045** | **0.027** |
| EBC nitrite | 0.111 | 0.097 | 0.084 | 0.071 | 0.036 | 0.001 | -0.034 |
| FeNO | 0.181 | 0.170 | 0.158 | 0.146 | 0.132 | 0.118 | 0.104 |
| EBC pH | 0.100 | 0.090 | 0.080 | 0.070 | 0.050 | 0.029 | 0.008 |
| MDA | 0.043 | 0.040 | 0.037 | 0.034 | 0.032 | 0.030 | 0.028 |
| **Systemic inflammation and oxidative stress** | **0.034** | **0.030** | **0.026** | **0.022** | **0.009** | **-0.004** | **-0.016** |
| Urinary 8-OHdG | 0.135 | 0.124 | 0.113 | 0.102 | 0.074 | 0.046 | 0.018 |
| Fibrinogen | 0.028 | 0.025 | 0.022 | 0.018 | 0.008 | -0.002 | -0.013 |
| WBC | -0.022 | -0.024 | -0.026 | -0.028 | -0.040 | -0.051 | -0.062 |
| RBC | -0.046 | -0.044 | -0.042 | -0.040 | -0.042 | -0.043 | -0.045 |
| Urinary MDA | 0.073 | 0.068 | 0.062 | 0.057 | 0.044 | 0.032 | 0.020 |
