# Supplementary material for: The Cardiopulmonary Effects of Ambient Air Pollution and Mechanistic Pathways: A Comparative Hierarchical Pathway Analysis
Source: PLoS One. 2014 Dec 12;9(12):e114913. doi: 10.1371/journal.pone.0114913 (PMC4264846; doi:10.1371/journal.pone.0114913)
Supplement: S1 Appendix — Description of air pollution measurement. (DOC) [file pone.0114913.s012.doc]

***Appendix S1.*** Description of air pollution measurement

In brief, a Quad-Channel ambient particulate sampler (TH-16A) was used in the field to collect a set of four Teflon and quartz filters at size cut of PM2*.*5, every 24 h between 10 and 10 a.m. Sulfate concentrations were analyzed from the quartz filters by ion chromatography. EC and OC were collected on heat-treated quartz fiber filters and measured using the NIOSH Method 5040 in a commercial laboratory (Rich et al. 2012). We also measured gaseous pollutants (sulfur dioxide, nitrogen dioxide, carbon monoxide, and ozone) using monitors that were calibrated and maintained following the manufacturer's protocols (Ecotech Ltd). We measured ambient temperature and relative humidity at the same site.
