# Supplementary material for: The Cardiopulmonary Effects of Ambient Air Pollution and Mechanistic Pathways: A Comparative Hierarchical Pathway Analysis
Source: PLoS One. 2014 Dec 12;9(12):e114913. doi: 10.1371/journal.pone.0114913 (PMC4264846; doi:10.1371/journal.pone.0114913)
Supplement: S2 Appendix — Description of biomarker measurement. (DOC) [file pone.0114913.s013.doc]

***Appendix S2.*** Description of biomarker measurement

*Autonomic function: including blood pressure, heart rate and heart rate variability (HRV),* was measured through 12-lead 3-channel MGY-S2 Electrocardiogram (ECG) Analysis Systems (ECG Lab 3.0, Meigaoyi Corp, Beijing) and the software program of the ECG systems (ECG lab 3.0, DM software Incorporation, Stateline, NV, US). The time-domain parameters we assessed included standard deviation of normal R-R intervals (SDNN) and root mean square of successive differences between adjacent normal cycles (rMSSD). We categorized the frequency spectrum into low frequency (LF) power (0.04 - 0.15 Hz) and high-frequency (HF) power (0.15 to 0.40Hz). We also computed the ratio of LF to HF. Other HRV parameters assessed using the ECG included very low frequency (VLF) power (0.003 to 0.04 Hz) and total power (total spectral power of all intervals up to 0.04 Hz). Blood pressure [systolic (SBP) and diastolic (DBP)] was measured using a manual sphygmomanometer after 5 mins of rest.

*Pulmonary inflammation and reactive oxidative stress* were assessed using exhaled breath condensate markers (EBC: pH, Nitrite, Malondialdehyde (MDA)) and fractional exhaled nitric oxide (FeNO). Exhaled breath condensates from tidal breathing were collected using a commercial breath-condensate collector (EcoScreen, Erich Jaeger, Germany). EBC pH and EBC nitrite was measured using an electronic pH meter and an HPLC system (Waters Model 2695, USA) with a UV detector (Waters Model 2996, USA), respectively(Huang et al. 2012). The method for analyzing MDA in EBC and urine samples used an HPLC system with fluorescent detection (Gong et al. 2013). Exhaled air from functional residual capacity was collected into a NO-impermeable aluminum foil bag (Huayuan Gas Center, China). The NO was analyzed by a NOx chemiluminescence analyzer (Model 42C NO-NO2-NOX Analyzer, Thermo) (Huang et al. 2012). EBC- 8-isoprostane was also measured, but not included in our analyses as there were many non-detects which would have decreased comparability across the different biomarkers.

*Markers of Thrombosis* [sP-selectin (sCD62P), CD40 Ligand (sCD40L), and Von Willebrand Factor (VWF)] and *systemic inflammation and oxidative stress* [plasma: Fibrinogen, red blood cells (RBC), and white blood cells (WBC); urine: MDA and 8-Hydroxy-2′-deoxyguanosine (8-OHdG)] were assessed. Concentrations of sCD62P and sCD40L in the plasma were measured using a enzyme-linked immunosorbent assay ([ELISA] Rapidbio). VWF was measured using a commercially available ELISA kit (Hushang Biotech). Plasma fibrinogen concentrations were analyzed using an automated ACL9000 analyzer. Blood cell counts were measured using standard automated clinical methods in the hospital.

We measured urinary 8-OHdG using an HPLC equipped with an electrochemical detector (Waters, Milford, MA). Though blood CRP was measured in this study, we have not included it in our analyses as there were a large number of non detects leading to the use of fraction detected as an outcome in previous publications (Rich et al. 2012). This would not have been a valid outcome in this particular analysis and thus this variable was dropped. Concentrations of urinary 8-OHdG were normalized by urinary creatinine concentrations. Urinary MDA was analyzed using the same method as used for the measurement of EBC MDA.
